# Assessment of the Potential Biological Activity of Low Molecular Weight Metabolites of Freshwater Macrophytes with QSAR

**DOI:** 10.1155/2016/1205680

**Published:** 2016-04-20

**Authors:** Evgeny A. Kurashov, Elena V. Fedorova, Julia V. Krylova, Galina G. Mitrukova

**Affiliations:** ^1^Institute of Limnology, Russian Academy of Sciences, Ulica Sevastyanova 9, Saint Petersburg 196105, Russia; ^2^Department of Ecological Security and Sustainable Development, Institute of Earth Sciences of Saint Petersburg State University, Ulica 10-ya Liniya 33–35, Saint Petersburg 199178, Russia; ^3^VVS Lab Inc., Ulica Dostoevskogo 44, Saint Petersburg 191119, Russia; ^4^Saint Petersburg State Chemical and Pharmaceutical Academy, Ulica Professora Popova 14, Saint Petersburg 197376, Russia

## Abstract

The paper focuses on the assessment of the spectrum of biological activities (antineoplastic, anti-inflammatory, antifungal, and antibacterial) with PASS (Prediction of Activity Spectra for Substances) for the major components of three macrophytes widespread in the Holarctic species of freshwater, emergent macrophyte with floating leaves,* Nuphar lutea* (L.) Sm., and two species of submergent macrophyte groups,* Ceratophyllum demersum* L. and* Potamogeton obtusifolius* (Mert. et Koch), for the discovery of their ecological and pharmacological potential. The predicted probability of anti-inflammatory or antineoplastic activities above 0.8 was observed for twenty compounds. The same compounds were also characterized by high probability of antifungal and antibacterial activity. Six metabolites, namely, hexanal, pentadecanal, tetradecanoic acid, dibutyl phthalate, hexadecanoic acid, and manool, were a part of the major components of all three studied plants, indicating their high ecological significance and a certain universalism in their use by various species of water plants for the implementation of ecological and biochemical functions. This report underlines the role of identified compounds not only as important components in regulation of biochemical and metabolic pathways and processes in aquatic ecological systems, but also as potential pharmacological agents in the fight against different diseases.

## 1. Introduction

Natural products have been used in folk medicine for many thousand years due to their biological origin, better ADME/TOX (absorption, distribution, metabolism, and excretion/toxicity) characteristics, and high chemical diversity. The importance, effectiveness, and prospects of herbal products use in medicine as antimicrobial agents are described in detail in [1].

Plants from terrestrial habitats have attracted substantial attention in bioprospecting investigations aimed at new bioactive compounds and drug discovery. The significance of available databases of medicinal plants and chemo- and bioinformatics tools for* in silico* drug discovery beyond the traditional use of folk Indian Medicine has been given adequate consideration in [2].

By contrast, the importance of freshwater macrophytes in this aspect has often been overlooked [3]. However, the existing evidence favors the view that naturally occurring compounds from aquatic ecosystems are of particular interest since many of them display important biological activities and possess antitumor, antibacterial, antimicrobial, antifouling, antifungal, pesticidal, phototoxic, HIV-inhibitory, and immunosuppressive properties [4].

Many of low molecular weight volatile organic compounds (VOCs) synthesized by aquatic plants are biologically active compounds and play a very important role in various processes that take place in aquatic ecosystems and influence the composition and development of aquatic biocenoses, actually structuring the environment [5]. However, they tend to remain undervalued biological resources. In particular, this underestimation is associated with very little information on VOCs of most species of aquatic macrophytes.

At present the component (chemical) composition of essential oils extracted from several species of aquatic macrophytes growing on the territory of Russia has been identified, and constituent patterns related to biosynthesis of VOCs in freshwater ecosystems have been revealed [6]. Recently close attention has been paid to freshwater macrophytes as they can be a source of anticancer and antioxidative natural products [3].

Currently, the number of identified VOCs in aquatic macrophytes exceeds several hundred compounds. They all merit attention in terms of evaluation of their biological activity. However, to begin with, it is advisable to identify the types of biological activity in the major components (>0.5% by the content of essential oil) of the whole spectrum of VOCs.

Secondary metabolites of aquatic macrophytes that have been identified can be characterized by a multiplicity of biological effects that are really difficult to discover and verify experimentally. At the same time, the identification of their biological activity by the SAR method, that is, predicting characteristics of the biological activity of the structures of chemical compounds [7], enables the further experimental study of substances identified in the natural primary products for medical, pharmacological, biological, or environmental purposes.

Method QSAR has shown its effectiveness in assessing the potential role of bioactive compounds in the aquatic environment in cases when other methods of studies were impossible or difficult [8]. Moreover, the combination of predictive bioactivities (QSAR methodology) with experimental data provides a most preliminary scientific approach allowing for expediently taking into consideration those compounds that require attention because of their particular values or prospects [9].

Prediction of Activity Spectra for Substances (PASS) (http://www.way2drug.com/) is software for the creation of SAR models based on MNA descriptors and modified Bayesian algorithm. PASS approach can be applied to so-called “drug-like” substances. It predicts several thousand types of biological activity, including pharmacological effects, mechanisms of action, toxic and adverse effects, interaction with metabolic enzymes and transporters, and the influence on gene expression [7]. PASS training set contains about 1 million compounds collected over many years from various publicly available sources and from commercially available sources. The knowledge base SAR Base is created in the process of training using PASS training set. SAR Base includes a biological activities dictionary and MNA descriptors dictionary, data and knowledge on the “structure-biological activity” relationships, and the database of structure of compounds from the training set with their biological activity spectra [7]. The structure of the compound is represented as a set of MNA descriptors. The results of the PASS procedure are output as a list of the differences between the probabilities, for each biological activity, of the compound to be active (*P*_*a*_) or to be inactive (*P*_*i*_). PASS does not predict if the compound will become a drug but helps to select the most prospective leads. The accuracy of PASS prediction for any type of activity is rather satisfactory when the number of compounds in the training set having the very kind of activity is more than 5 [7].

The use of* in silico* methods for drug discovery in natural products has increased during the previous decade [10]. The appearance of new chemo- and bioinformatics methods along with a growing range of OMICs data and the data on phytochemical structures has opened vast perspectives in the study of the pharmacological activity of plant preparations.

This paper focuses on the assessment of the spectrum of biological activities with PASS for the major components of three macrophytes widespread in the Holarctic: emergent macrophyte with floating leaves,* Nuphar lutea* (L.) Sm., and two species of submergent macrophyte group,* Ceratophyllum demersum* L. and* Potamogeton obtusifolius* (Mert. et Koch).

In this regard, the purpose of the present study was to forecast the spectrum of biological activities with the QSAR method for the identified major components of the essential oil of* N. lutea*,* C. demersum*, and* P. obtusifolius* and to identify promising compounds for further experimental research designed for medical, pharmaceutical, cosmetic, and environmental purposes.

Out of the estimated spectrum of biological activity (>250), special attention in this paper is given to the results of predicting the probability of antineoplastic, anti-inflammatory, antifungal, and antibacterial activities of metabolites of the studied plant species that allows evaluating the prospects of using the identified VOCs, particularly, in medicine, pharmacology, and environmental biotechnology.

## 2. Material and Methods

### 2.1. Plant Materials

The composition of VOCs of macrophytes* N. lutea*,* C. demersum*, and* P. obtusifolius* was investigated in the plant material which was collected in the following habitats: (i) oligotrophic Lake Suuri (Karelian Isthmus, Leningrad Oblast, N 61°07.859′, E 29°55.076′) with still waters and sticky silt bottom, (ii) an area in the estuary of the Volkhov River (Volkhov Bay, Lake Ladoga, N 60°07.139′, E 32°19.566′) with fast waters and silty sand bottom, and (iii) ponds of the Victory Park (St. Petersburg, N 60°52.025′, E 30°19.91′) with small depths and silt bottom.

Sampled plants were washed free of debris and air-dried in a closed and darkened room without direct sunlight. Before distillation, the dried plant material was ground to a powder in a Waring BB_25ES blender (Waring, United States).

### 2.2. Isolation of Oils

Air-dried plants (15–40 g) were placed in a round-bottomed flask and steam-distilled with Clevenger apparatus [11]. Hydrodistillation was carried out for 8 hours. The essential oil samples were extracted with hexane. The extracts were stored in a freezing chamber (−18°C) prior to GC-MS analyses.

### 2.3. GC-MS Analysis

The composition of VOCs was analyzed in the hexane extracts using a TRACE DSQ II gas chromatography-mass spectrometer (Thermo Electron Corporation) equipped with a quadrupole mass analyzer and TRACE TR_5MS GC Column (15 m, 0.25 mmID, and 0.25 *μ* film). Helium served as a carrier gas. Mass spectra were registered in the scan mode for the whole mass range (30–580 amu) in a programmed temperature regime (oven temperature was held at 35°C for 3 min and then programmed to increase to 60°C at a rate of 2°C/min, was kept constant at 3 min and then programmed to increase to 80°C at a rate of 2°C/min, was kept constant at 3 min and then programmed to increase to 120°C at a rate of 4°C/min, was kept constant at 3 min and then programmed to increase to 150°C at a rate of 5°C/min, and was kept constant at 3 min and then programmed to increase to 240°C at a rate of 15°C/min and then held isothermal for 10 min). The discovered VOCs of the essential oils were identified by matching their mass spectra with those from the NIST_2008 and the Wiley mass spectral libraries. The identification of compounds was confirmed by Kovats retention indices obtained from a series of straight chain alkanes (C7–C30). Quantitative analysis was performed with decafluorobenzophenone and benzophenone as internal standards.

### 2.4. PASS Analysis

*In silico* predictions were carried out using the retrained version of PASS for 72 VOCs that have been identified as major components of freshwater macrophytes mentioned above. PASS is a software product for the prediction of biological activity spectra for organic compounds on the basis of their structural formula. As input information PASS 2014 Refined used the one on the structural formula of the molecule represented in a file format Molfile (Accelrys, Inc., http://accelrys.com/) (for the same structure) or in a file format SDfile (Accelrys, Inc., http://accelrys.com/) for sampling structures. PASS software estimates the predicted activity spectrum of a compound as probable activity (*P*_*a*_) and probable inactivity (*P*_*i*_). Prediction of this spectrum by PASS is based on SAR analysis of the training set containing about one million compounds showing more than 7000 biological activities.

The PASS approach has been previously described in detail in [7], and there are also many publications where PASS predictions were confirmed by subsequent synthesis and biological testing [12–14].

The training set PASS as “active” compounds are considered compounds having the quantitative characteristics of activity better than 10^−4 ^M. All the other compounds which are less active or their activity is unknown are considered as “inactive.” The PASS user obtains output information as a list of predicted types of activity with the estimated probability for each type of activity: “to be active” *P*_*a*_ and “to be inactive” *P*_*i*_. The probabilities *P*_*a*_ and *P*_*i*_ also indicate the estimated probabilities of first-kind and second-kind errors, respectively [7]. Being probabilities, *P*_*a*_ and *P*_*i*_ values vary from 0.000 to 1.000 and, in general, *P*_*a*_ ≠ *P*_*i*_ = 1, since these probabilities are calculated independently. For *P*_*a*_ > 90%, we risk missing about 90% of actually active compounds but the probability of false-positive predictions is insignificant. For *P*_*a*_ > 80%, we already miss only 80% of the active compounds but the probability of false-positive predictions will be higher, and so forth. Finally, for *P*_*a*_ = *P*_*i*_, the probabilities of false-positive and false-negative errors will be equal [7]. It should be noted that the probability *P*_*a*_ primarily reflects the similarity of the structure of a given molecule to the structures of molecules of the most typical active compounds in the corresponding subset of the training set. Thus, there is no direct correlation of the values of *P*_*a*_ with quantitative activity characteristics. A really active molecule possessing molecular structure atypical for the training set may have a low *P*_*a*_ value in the prediction, perhaps even *P*_*a*_ < *P*_*i*_. Another important aspect of interpreting the prediction results is related to novelty of the analyzed compound. If we limit ourselves only to activity types predicted with the highest values of *P*_*a*_, the compounds selected by the prediction may prove to be analogs of known pharmacological agents. For example, when *P*_*a*_ > 0.7, the chances of finding experimental activity are rather high but the compounds found may be close structural analogs of known drugs. If we select in the range 0.5 < *P*_*a*_ < 0.7, the chances for detecting experimental activity will be lower but the compounds will be less similar to known pharmaceutical agents. For *P*_*i*_ < *P*_*a*_ < 0.5, the chances of detecting experimental activity will be even lower, but if the prediction is confirmed, the compound found may prove a parent compound for a new chemical class for the biological activity examined [7].

The PASS estimations of biological activity spectra of new compounds are based on the Structure-Activity Relationships data and knowledge base (SAR Base), which accumulates the results of the training set analysis. The in-house developed general PASS training set currently includes more than million known biologically active substances (drugs, drug candidates, leads, and toxic compounds). In the specific version of the PASS (if you are using the same version of the program) *P*_*a*_ calculated values depend only on the formula of test compound. So, the results will be the same regardless of the number of runs. PASS does not perform the pairwise comparisons between the structure of the new compounds and the structure of compounds from the training set and just calculated probability of belonging to the class of “active” and “inactive” compounds, respectively. Therefore, the structure of the “standard agents” can be represented as “generalized image of matter” in space of MNA descriptors. It is, therefore, not possible to present such image to the reader.

## 3. Results and Discussion

A total of 72 VOCs classified as major components of the essential oil (>0.5% of the entire spectrum of VOCs) were identified for* N. lutea*,* C. demersum*, and* P. obtusifolius*. In order to predict the biological activity profiles the structural formulas of the test 72 major compounds were presented in the form of SDF-file and then used in the virtual screening based on commercially available software product, PASS 2014 Refined.

Assessment of the probability of biological activity of the identified major components of the essential oils of the studied plants is shown in Table 1. Only compounds (63 out of 72) for which the values of the probability of the existence of any of the activities exceed 0.4 were included in Table 1.

Prediction results of activity spectra for test compounds (Table 1) showed that there was high probability of anti-inflammatory, antineoplastic, antibacterial, and antifungal activities for these compounds. The number of substances in the training set and accuracy of prediction (%) for pharmacological effects obtained for test components by PASS 2014 Refined are given in Table 2.

The results, presented in Table 1, indicate significant prospects for experimental studies of the identified VOCs of aquatic macrophytes. For forty compounds the predictions of some or at least one of the four biological activities were above 0.7 (Table 1).

The predicted value of anti-inflammatory or antineoplastic activities with probability above 0.8 was observed for twenty compounds, namely, (2Z,4Z)-hepta-2,4-dienal; 2-phenylacetaldehyde; (3E,5E)-octa-3,5-dien-2-one; 2,6-dimethylcyclohexan-1-ol; geranylacetone; *α*-muurolene; *β*-ionone; *β*-eudesmol; *α*-eudesmol; androstanol; sandaracopimaradiene; biformen; 5*α*-androstan-16-one; muquketone; rimuen; kaurene; manool; (9Z,12Z)-octadeca-9,12-dienoic acid; 8-(2,5,5,8A-tetramethyl-1,4,4A,5,6,7,8,8A-octahydro-1-naphthalenyl)-6-methyl-5-octen-2-ol; and (22E)-3*α*-ergosta-14,22-dien-5*β*-ol acetate. The same compounds were also characterized by high probability of antifungal and antibacterial activity (Table 1), indicating a potentially high significance of these VOCs in ecological and biochemical interaction of higher aquatic plants with microbial and fungal populations in aquatic ecosystems, as well as high pharmacological potential of these compounds. The obtained data on the environmental importance of major VOCs are consistent with the data available in corresponding literature [6].

There is little information in literature that discovered that major constituents of aquatic plants possess antineoplastic, anti-inflammatory, antifungal, and antibacterial properties.

For *β*-eudesmol it is the antifungal [15] and antibacterial [16] properties that were described. According to the literature, manool demonstrates different types of biological activities (antioxidant, antimicrobial, and antifungal) that are important in terms of ecological interactions [17–20]. Furthermore, the other properties (platelet aggregation inhibitory activity, anti-inflammatory activity, and cytostatic activities against human malignant cell strains) are promising for medicine and pharmacology [21, 22].

Zhu and his coauthors [23] have shown that 2-phenylacetaldehyde exhibited a significant antifungal activity through the inhibition of the mushroom tyrosinase, but it lacked an antibacterial activity. Antibacterial property of *β*-ionone manifests itself in the fact that it inhibits the photosynthetic system of* Microcystis aeruginosa* [24]. It is reported [25] that good antifungal potential of violet oil mainly against all tested pathogenic fungi can probably be attributed to the high content of *α*-ionone and *β*-ionone for which there are data of the antifungal activity [26].

According to the results of PASS prediction, hexanol has not showed high antifungal and antibacterial activities (0.45 and 0.33, resp.) (Table 1). However, the antifungal and antibacterial properties of hexanol are described in [27].

*Flourensia oolepis* Blake (Asteraceae) essential oil with high content of muurolene had repellent and toxic effects on* Tribolium castaneum* Herbst (Coleoptera: Tenebrionidae) adults, acting as a contact toxin [28]. The leaf oil of* Lindera strychnifolia* (Sieb. and Zucc.) F. Villars (Lauraceae) containing muurolene in abundance showed the strongest cytotoxicity on the cancer cell lines [29].

As observed in [30], the sandaracopimaradiene showed a fairly strong activity against the tested Gram-positive bacteria (*Bacillus subtilis *and* Streptococcus pyogenes*) as well as a good activity against some Gram-negative bacteria (*Proteus vulgaris*,* Shigella dysenteriae*, and* Pseudomonas solanacearum*). The activity against the tested fungi is less pronounced, except for the yeast [30].

At the same time it was demonstrated that sandaracopimaradiene and its derivatives, what are known as phytoalexins, are antifungal compounds in the plant families, Leguminosae and Rosaceae, and in rice,* Oryza sativa*. Phytoalexins play an important role in the resistance of rice plants against fungus* Pyricularia oryzae *[31]. Also, sandaracopimaradiene is antitrichomonas agent, and it has been isolated from the leaves of* Tetradenia riparia *[32].

**Figure 1:**
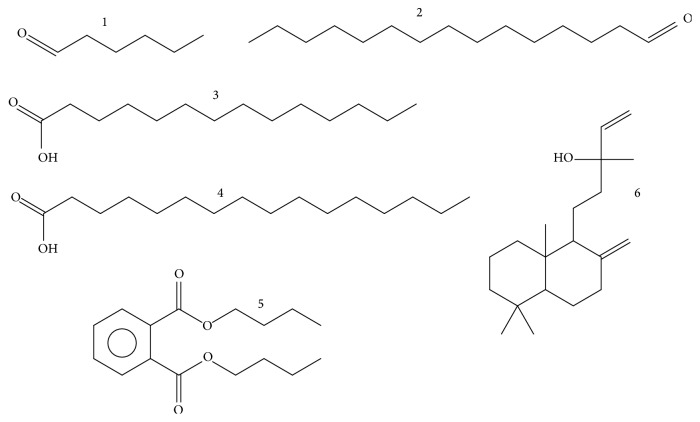
Structure of hexanal (1), pentadecanal (2), tetradecanoic acid (3), hexadecanoic acid (4), dibutyl phthalate (5), and manool (6).

Six VOCs, namely, hexanal, pentadecanal, tetradecanoic acid, dibutyl phthalate, hexadecanoic acid, and manool (Figure 1), were a part of the major components of all three studied plants, indicating their high ecological significance and certain universalism in their use by various species of water plants for the implementation of ecological and biochemical functions.

Biological activities of the secondary metabolites of aquatic macrophytes discovered with PASS prediction suggest that some of these above-mentioned compounds seem to be agents involved in chemical defense of plants from pathogens and herbivorous invertebrates and fishes in the same manner as in terrestrial ecosystems [33].

It was observed that* C. demersum* comprises the largest number of major components of VOCs (45 out of 63) with the probability of the four types of studied biological activities above 0.4, while* N. lutea* and* P. obtusifolius* comprise 15 and 21 components, respectively. This may indicate greater ecological plasticity and activity of* C. demersum*, for example, in the process of allelopathic interactions in aquatic ecosystems. This fact also makes* C. demersum* more promising for the search of effective compounds-allelochemicals in the development of biotechnology of regulation and suppression of cyanobacteria with the use of natural algaecides [34].

The highest value in anti-inflammatory activity (about 0.9) was observed in (3E,5E)-Octa-3,5-dien-2-one; *β*-eudesmol; rimuen; kaurene; and manool and that in antineoplastic activity was observed in *β*-eudesmol; androstanol; sandaracopimaradiene; biformen; 5*α*-androstan-16-one; rimuen; kaurene; and (22E)-3*α*-ergosta-14,22-dien-5*β*-ol acetate.

The results from [35, 36] add considerable support for the statement that 5*α*-androstan-16-one and similar compounds from aquatic macrophytes can serve as perspective anticancer agents which can inhibit proliferation of cancer cell lines. There are also the immediate and long-term prospects for use of these constitutes as agents against the resistant fungal strains, combined with systemic fungal infections [37].

As reported in [38] the kaurenes showed activity against the seven cancer cell lines tested, with the highest overall activity being observed for the prostate cancer cells. Because of the biological activities, separate VOCs and the oils from water macrophytes may be proposed as agents in food preservation. This use of the oils was described in [39].

Based on the results of the PASS prediction previously imperfectly understood types of biological activities (antineoplastic, anti-inflammatory, antifungal, and antibacterial) of freshwater macrophytes can be recommended to the experimental study.

## 4. Conclusions

In the course of the research the predicted values of biological activities (antineoplastic, anti-inflammatory, antifungal, and antibacterial) for 72 major components of aquatic macrophytes* C. demersum*,* P. obtusifolius*, and* N. lutea* have been obtained.

These results form the basis for further experimental research of the most promising natural products of the VOCs of aquatic macrophytes for medical, pharmacological, biological, or environmental purposes. Other types of biological activities of the identified major VOCs, promising for environmental or pharmacological purposes, should also be evaluated; and experimental evaluation of the biological activities of individual compounds of aquatic vegetation needs to be performed in accordance with the results obtained during the research with the use of the QSAR method.

## Figures and Tables

**Table 1:** The estimated probability (*P*_*a*_) of the existence of biological activity (anti-inflammatory (AI), antifungal (AF), antibacterial (AB), and antineoplastic (AN)) of identified major components (>0.5%) of essential oil of *N. lutea* (NL), *C. demersum* (CD), and *P. obtusifolius* (PO); IK: Kovats retention index.

Number	Major constituents	IK	AI	AF	AB	AN	Plant
1	Hexan-2-one	790	0.697	0.421	0.317	0.557	NL
2	Hexanal	796	0.271	0.524	0.436	0.226	NL, PO, CD
3	***(E)-Hex-2-en-1-ol***	***857***	***0.659***	***0.545***	***0.435***	***0.716***	***PO***
4	Hexan-1-ol	865	0.659	0.447	0.33	0.532	NL, CD
5	Heptan-2-one	906	0.697	0.421	0.317	0.557	CD
6	***(E)-Hept-4-enal***	***913***	***0.706***	***0.591***	***0.487***	***0.383***	***CD***
7	Heptanal	915	0.271	0.524	0.436	0.226	CD
8	***(2Z,4Z)-Hepta-2,4-dienal***	***932***	***0.801***	***0.523***	***0.412***	***0.761***	***CD***
9	***Oct-1-en-3-ol***	***994***	***0.783***	***0.656***	***0.413***	***0.748***	***CD***
10	***6-Methylhept-5-en-2-one***	***1001***	***0.785***	***0.531***	***0.478***	***0.758***	***CD***
11	4,7,7-Trimethyl-8-oxabicyclo[2.2.2]octane; [1,8-cineol, eucalyptol]	1033	0.327	0.216	0.478	0.243	CD
12	***2-Phenylacetaldehyde***	***1050***	***0.811***	***0.228***	***0.297***	***0.172***	***CD***
13	***(3E,5E)-Octa-3,5-dien-2-one***	***1104***	***0.881***	***0.584***	***0.445***	***0.772***	***CD***
14	***2,6-Dimethylcyclohexan-1-ol***	***1110***	***0.655***	***0.571***	***0.513***	***0.848***	***CD***
15	2,6,6-Trimethylcyclohexa-1,3-diene-1-carbaldehyde; [safranal]	1202	0.444	0.266	0.282	0.697	CD
16	***2,6,6-Trimethyl-1-cyclohexene-1-carbaldehyde; [β-cyclocitral]***	***1224***	***0.438***	***0.31***	***0.303***	***0.783***	***CD***
17	***6-Hydroxy-4,4-dimethyl-3H-chromen-2-one***	***1327***	***0.684***	***0.319***	***0.299***	***0.726***	***CD***
18	6,10-Dimethylundecan-2-one	1422	0.657	0.512	0.372	0.546	CD
19	***(E)-4-(2,6,6-Trimethylcyclohex-2-en-1-yl)but-3-en-2-one; [α-ionone]***	***1437***	***0.565***	***0.588***	***0.495***	***0.717***	***CD***
20	*** (5E)-6,10-Dimethylundeca-5,9-dien-2-one; [geranylacetone]***	***1469***	***0.817***	***0.594***	***0.47***	***0.808***	***CD***
21	***4,7-Dimethyl-1-propan-2-yl-1,2,4a,5,6,8a-hexahydronaphthalene; [α-muurolene]***	***1485***	***0.751***	***0.511***	***0.532***	***0.818***	***CD***
22	***(E)-4-(2,6,6-Trimethylcyclohexen-1-yl)but-3-en-2-one; [β-ionone]***	***1494***	***0.752***	***0.376***	***0.335***	***0.844***	***CD***
23	4-(2-Methyl-3-oxocyclohexyl)butanal	1525	0.444	0.589	0.495	0.471	CD
24	***8a-Methyl-3,4,4a,5,6,7-hexahydro-2H-naphthalene-1,8-dione***	***1530***	***0.457***	***0.28***	***0.262***	***0.732***	***CD***
25	2-[(2R,4aR,8aR)-4a,8-Dimethyl-2,3,4,5,6,8a-tetrahydro-1H-naphthalen-2-yl]propan-2-ol	1536	0.69	0.462	0.449	0.624	CD
26	2-(6,10-Dimethylspiro[4.5]dec-9-en-3-yl)propan-2-ol; [agarospirol]	1604	0.598	0.282	0.467	0.449	CD
27	[2,4,4-Trimethyl-3-(2-methylpropanoyloxy)pentyl] 2-methylpropanoate	1609	0.334	0.459	0.203	0.148	CD
28	***2-[(3S,5R,8S)-3,8-Dimethyl-1,2,3,4,5,6,7,8-octahydroazulen-5-yl]propan-2-ol; [guaiol]***	***1638***	***0.672***	***0.31***	***0.379***	***0.760***	***CD***
29	***2-[(2R,4aS)-4a,8-Dimethyl-2,3,4,5,6,7-hexahydro-1H-naphthalen-2-yl]propan-2-ol; [γ-eudesmol]***	***1639***	***0.738***	***0.331***	***0.42***	***0.731***	***CD***
30	***2-[(2R,4aR,8aS)-4a-Methyl-8-methylidene-1,2,3,4,5,6,7,8a-octahydronaphthalen-2-yl]propan-2-ol; [β-eudesmol]***	***1659***	***0.869***	***0.524***	***0.52***	***0.879***	***CD***
31	***2-[(2R,4aR,8aR)-4a,8-Dimethyl-2,3,4,5,6,8a-hexahydro-1H-naphthalen-2-yl]propan-2-ol; [α-eudesmol]***	***1663***	***0.818***	***0.479***	***0.487***	***0.804***	***CD***
32	***2-(4a,8-Dimethyl-2,3,4,5,6,7,8,8a-octahydro-1H-naphthalen-2-yl)propan-2-ol; [dihydro-β-eudesmol]***	***1666***	***0.794***	***0.505***	***0.465***	***0.747***	***CD***
33	2-(3,8-Dimethyl-1,2,3,3a,4,5,6,7-octahydroazulen-5-yl)propan-2-ol; [bulnesol]	1675	0.676	0.431	0.426	0.660	CD
34	Heptadecane	1700	0.583	0.378	0.31	0.316	CD
35	Pentadecanal	1732	0.271	0.524	0.436	0.226	NL, PO, CD
36	5,5-Dimethyl-2-propan-2-ylcyclohexane-1-carboxylic acid	1749	0.553	0.483	0.338	0.526	CD
37	***Tetradecanoic acid***	***1777***	***0.709***	***0.48***	***0.384***	***0.563***	***NL, PO, CD***
38	Octadecane	1800	0.583	0.378	0.31	0.316	CD
39	***(3S,5S,8S,9S,10S,13S,14S)-10,13-Dimethyl-2,3,4,5,6,7,8,9,11,12,14,15,16,17-tetradecahydro-1H-cyclopenta[a]phenanthren-3-ol; [androstanol]***	***1839***	***0.794***	***0.587***	***0.455***	***0.858***	***NL***
40	6,10,14-Trimethylpentadecan-2-one; [phytone]	1845	0.657	0.512	0.372	0.546	NL, PO
41	***Pentadecanoic acid***	***1884***	***0.709***	***0.347***	***0.384***	***0.563***	***NL***
42	*** (4aS,4bS,7S,10aS)-7-Ethenyl-1,1,4a,7-tetramethyl-3,4,4b,5,6,9,10,10a-octahydro-2H-phenanthrene; [sandaracopimaradiene; pimaradiene]***	***1916***	***0.82***	***0.411***	***0.362***	***0.846***	***NL***
43	***4b,8,8-Trimethyl-5,6,7,8a,9,10-hexahydrophenanthren-3-ol***	***1920***	***0.749***	***0.483***	***0.357***	***0.760***	***PO***
44	Nonadecane	1926	0.583	0.378	0.31	0.316	CD
45	*** 4,4,8a-Trimethyl-7-methylidene-8-[(2E)-3-methylpenta-2,4-dienyl]-2,3,4a,5,6,8-hexahydro-1H-naphthalene; [biformen]***	***1946***	***0.822***	***0.707***	***0.623***	***0.896***	***PO, CD***
46	***5α-Androstan-16-one***	***1958***	***0.786***	***0.437***	***0.339***	***0.860***	***PO***
47	Dibutyl benzene-1,2-dicarboxylate; [dibutyl phthalate]	1961	0.525	0.365	0.256	0.237	NL, PO, CD
48	*** (E)-6-Methyl-8-(2,6,6-trimethylcyclohexen-1-yl)oct-5-en-2-one; [muquketone]***	***1963***	***0.796***	***0.497***	***0.457***	***0.843***	***PO***
49	*** (Z)-Hexadec-11-enoic acid***	***1977***	***0.769***	***0.565***	***0.434***	***0.645***	***CD***
50	***Hexadecanoic acid***	***1987***	***0.709***	***0.48***	***0.384***	***0.563***	***NL, PO, CD***
51	*** (2S,4aS)-2-Ethenyl-2,4a,8,8-tetramethyl-3,4,4b,5,6,7,10,10a-octahydro-1H-phenanthrene; [rimuen]***	***2035***	***0.878***	***0.52***	***0.361***	***0.850***	***PO***
52	***Kaur-16-ene; [kaurene]***	***2042***	***0.873***	***0.508***	***0.571***	***0.896***	***PO, CD***
53	***5-[(1S,4aS,8aS)-5,5,8a-Trimethyl-2-methylidene-3,4,4a,6,7,8-hexahydro-1H-naphthalen-1-yl]-3-methylpent-1-en-3-ol; [manool]***	***2047***	***0.846***	***0.748***	***0.621***	***0.821***	***NL, PO, CD***
54	***Methyl (5E,8E,11E)-heptadeca-5,8,11-trienoate***	***2065***	***0.786***	***0.556***	***0.382***	***0.577***	***NL***
55	Heneicosane	2100	0.583	0.378	0.31	0.316	PO, CD
56	***(9Z,12Z)-Octadeca-9,12-dienoic acid***	***2146***	***0.829***	***0.574***	***0.443***	***0.653***	***NL***
57	2-Ethylhexyl (E)-3-(4-methoxyphenyl)prop-2-enoate	2156	0.659	0.509	0.292	0.262	NL
58	***(E,7R,11R)-3,7,11,15-Tetramethylhexadec-2-en-1-ol; [phytol]***	***2174***	***0.696***	***0.66***	***0.527***	***0.760***	***PO, CD***
59	Tricosane	2300	0.583	0.378	0.31	0.316	PO
60	***3α-Hydroxy-5β-pregnan-20-one; [pregnanolone]***	***2318***	***0.736***	***0.568***	***0.431***	***0.799***	***PO***
61	***8-(2,5,5,8A-Tetramethyl-1,4,4A,5,6,7,8,8A-octahydro-1-naphthalenyl)-6-methyl-5-octen-2-ol***	***2364***	***0.798***	***0.778***	***0.651***	***0.812***	***PO***
62	***5-[2-(3-Furyl)ethyl]-1,4a-dimethyl-6-methylenedecahydro-1-naphthalenecarboxylic acid***	***2471***	***0.741***	***0.601***	***0.559***	***0.772***	***PO***
63	***(22E)-3α-Ergosta-14,22-dien-5β-ol acetate***	***2740***	***0.742***	***0.418***	***0.281***	***0.887***	***PO***

Notes: constituents with *P*_*a*_ > 0.7 are highlighted in bold italics. Trivial and commonly accepted names of some compounds are given in square brackets.

**Table 2:** List of pharmacological effects projected for natural compounds by PASS.

Effect name	Number of active compounds	Accuracy of prediction %
Anti-inflammatory	5983	84.7
Antineoplastic	30811	84.7
Antibacterial	8342	92.2
Antifungal	2470	92.5
